# Diphtheria

**Published:** 1860-10

**Authors:** A. K. Van Horn


### ARTICLE 4.

### DIPHTHERIA.

BY DR. A. K. VAN HORN.

Some account of the so-called disease, as it prevailed in the Northwest, and especially in Rock Run, during the summer and autumn of 1860.

Late in the Spring, a disease characterized by exudation and ulceration of the fauces, made its appearance in the village of Rock Grove, ten miles distant, which was christened and treated by the Physicians, as diphtheria, or Euro pean fever.

It prevailed chiefly among children; but in some instances attacked persons above the age of puberty. The mortality was very high, and the disease ran its course in from two days to a fortnight.

Sporadic cases of a mild character occurred in our practice during the summer months, but not till some time in August did the disease assume a malignant form. A description of one or two of these cases will give a good idea of the pathology, as the symptoms of all are very similar, and afford a foundation for some deductions, going far to prove, either that many practitioners err in the diagnosis of the disease, or that diphtheria is identical with scarlatina.

Case 1.—John R——b, a lad about 11 years of age, of remarkably nervous temperament, was attacked about August 10th. He complained of languor, weariness, pain in the back and llmbs, intense headache, and nausea, followed by violent vomiting and purging. His skin was dry and hot, pulse frequent and feeble, was delirious at night. He complained of soreness of the fauces and stiffness of the jaws. On inspection, found the mucous membrane congested, and the fauces partially covered with a thin curdy exudation. The tongue presented a yellowish fur, through which the papillar projected. On the second day of the fever, a rash made its appearance, first on the breast, and rapidly spreading, till it covered the whole body. It was at first punctated, but soon became a universal blush. The fever continued, but rapidly assumed a typhous character. The pulse grew more and more frequent, dark sordes encrusted the teeth, but owing probably, to the inflammation of the fauces extending to the cerebral membranes, the delirium continued of a very violent character. The rash grew dark and livid, the throat became a mass of ulcers, the mouth had a black, mahogany hue, a fetid sanies flowed from the nostrils; dark, gumous blood oozed from fissures in the lips, gangrenous eschars formed on exposed parts, the skin hung in shreds from his limbs, rapid and stertous breathing, with inability to swallow, finished the gloomy picture. Collape ensued, with cool skin, ghastly features, and death followed, in a week from the attack.

CASE 2.—A few days after our first visit, a second child showed symptoms of illness. The usual premonitory features of fever were present, with the addition of vomiting and purging. On examination, the fauces appeared congested, with a trace of exudation, which rapidly grew thicker, as the disease advanced. Within three days a rash appeared, at first of a bright red color, but as typhous symptoms set in, changing to a livid hue. Instead of the marked delirium of the first case, there was stupor. Stertorous breathing, fetid flow from the nosirils, putrid ulcers in the throat, inability to swallow, and death in a week.

In the milder cases, the disease ran its course in the following manner, with, of course, slight variations: The usual preliminaries of fever, such as languor, rigors, pains in the back and limbs, followed by a frequent pulse, hot skin, etc., were invariably present. In addition, vomiting and purging usually occurred, accompanied with soreness of the throat, externally and internally, distinguishing it from cholera morbus, which, in some respects, it closely imitated. An examination of the fauces at this stage, showed congestion, and a slight layer of plastic lymph. Within three days a well-defined rash appeared, the exudation grew thicker, and the swab brought away masses of it in a decayed condition. True ulcers sometimes formed in the arches, discharging a fetid pus. The eyes grew red but not watery; cough sometimes harrassed the patient; the tongue was apt to lose its coating early in the disease, becoming pointed, smooth, deep red and glossy.

In four or five days from its first appearance, the rash began to fade, the false membrane was thrown off, and the soreness diminished; the fever declined and the appetite returned. Unpleasant sequealla were apt to follow. In one instance dropsy carried off the patient soon after apparent recovery.

An anomalous feature occurred in another. His throat had been deeply ulcerated; the veins undoubtedly laid bare, and possibly their coats corroded. A slight cough ruptured a blood-vessel, and in five minutes, not less than a quart of blood flowed from the sufferer's mouth. Luckily, the discharge ceased with syncope, leaving the little patient exhausted but apparently much relieved of a swelling situated below the right parotid gland. The bleeding, however, continued again and again, and the child now lies dropsical and anemic to the last degree.

The following plan of treatment was adopted, which has so far proved successful, except in two cases of extraordinary violence. Believing the disease to be constitutional, the effect of a specific poison of a typhous nature, and the exudation but a symptom, like the eruption, and having a certain course of inception, maturity and decline, regardless of medicine, except to modify or diminish the violence of the symptoms, the following indications seemed to be presented:

1st. To allay vomiting and purging by means calculated to diminish the intestinal irritation.

2d. To moderate the fever by diaphoretics, and thus favor the eruption.

3d. To support the vital powers, from the beginning.

4th. Local applications to counteract the exudation, and prevent the formation of gangrenous ulcers.

Calomel, from its soothing effect, would seem peculiarly fitted to fulfill, in part, the first indication; but from its specific effect on the mucous membrane, and ill results in a "malignant condition of the system," its use was discarded, and the following recipe given, with uniform success.

℞ Opii. grs. ¼,

Sub. nitrate bismuth, grs. 3.

Administered in 3 or 4 minims of tinc. camphorae.

To fulfill the second and third indications, the following ℞ was used:

Pulvus Dov., grs. 3,
Sul. quinine, gr. ¼.

Local applications, are of the highest value, and nitrate of silver stands unrivalled, for its antophlogistic and alterative effect, when applied to the mucous membrane. In mild cases, in our hands, it has operated like a charm, and in the desperate, with deep ulcers and dark discoloration, it has ameliorated the symptoms and eased the suffering, though it might not protract life. It may be used in any proportion, from two grs. to the oz. of water, up to the solid stick.

A fifth indication might have been made, to produce an alterative effect on the system, and prevent the tendency to gangrenous ulceration. Chlorate of potash is recommended as modifying the state of the blood, changing it from a dark hue to a bright red. In the more violent forms of the disease, the blood is depraved, as the dark sordes and livid rash proves and whether this is a *cause* or *effect* of the poison, the chlorate may prove useful. Our experience with it is too limited to hazard an opinion.

As a wash to correct the fetor of the ulcers, the chlorate, in the proportion of two to four drachms to a pint of water, is an admirable application, and a small quantity of the same injected into the nostrils will often alleviate the filthy flow.

During the course of the disease the room should be well ventilated, and all discharges carefully removed. An occasional sponging of the patient with tepid water, will conduce much to his comfort.

From our experience of the disease, which extends to some thirty cases, of all grades of violence, from the slightest soreness of the fauces up to the most malignant form, we have arrived at the following conclusion; that most, if not all, the cases occurring in our region, diagnosed and treated by other physicians as diphtheria, or Europeon fever, have been one or the other of the three forms of scarlatina.

When the epidemic first reached us, we were carried away with *diphtheretism*, and pronounced the first cases as attacked with the new diseases; but a very short acquaintance made us *doubt*, and a *longer*, to recognise an old scourge, veiled in uncommonly malignant symptoms. Arguments to support our opinion will occupy a few lines:

1st. The symptoms of inception, maturity, and decline, accord precisely with those seen in former years in scarlatina.

2d. Descriptions of scarlatina, by the best authors, accord with our cases in every particular.

3d. Persons are exempt from an attack who have previously had scarlatina.

4th. The same sequela follow in its train.

From these, and other arguments, perceptible yet scarcely tangible, we are forced to conclude that there may be a distinct disease called diphtheria; but that the affection we have described is scarlatina, though differently termed by physicians of respectability.

---

### VITALITY OF TOADS.

Towards the year 1850, a workman in the neighborhood of Blois, (France,) struck a mass of silex with his pickaxe, and saw a live toad jump out of the stone. At that period doubts were entertained as to the supposed indefinite vitality of toads, which had been deduced from the above-mentioned fact; and M. Dumeril showed that toads, having cartilaginous ribs, could introduce the whole body where the head would enter. These animals are fond of slipping thus into very small apertures and crevices; and hence the error about their vitality when confined within stone. M. Milne Edwards' father, in order to bring the matter under direct experiment, buried some toads in plaster, and where, from the porosity of the plaster, or the existence of crevices, air could pentrate, the creatures lived several weeks, and even months; one, in fact, lived thus for *eighteen months*, this being the only authentic fact of the kinds. When, however, the plaster had neither porosity nor crevices, the animals were always found dead and dried up.

At a late meeting of the Academy of Sciences of Paris, the members present had an opportunity of breaking up masses of plaster, in which M. Seguin had imprisoned a viper and a toad in 1852. Both animals were found dead, and evidently dried up for a number of years.—*Lancet.*

